# Proteomics reveals that quinoa bioester promotes replenishing effects in epidermal tissue

**DOI:** 10.1038/s41598-020-76325-6

**Published:** 2020-11-10

**Authors:** Amanda C. Camillo-Andrade, Marlon D. M. Santos, Juliana S. G. Fischer, Bruna B. Swinka, Bruna Bosquetti, Desirée C. Schuck, Marcia R. Pincerati, Marcio Lorencini, Paulo C. Carvalho

**Affiliations:** 1 Master Program in Industrial Biotechnology, Positivo University, R. Prof. Pedro Viriato Parigot de Souza, 5300, Curitiba, Paraná Brazil; 2 Laboratory for Structural and Computational Proteomics, Carlos Chagas Institute, Fiocruz - Paraná, R. Professor Algacyr Munhoz Mader 3775, Curitiba, PR Brazil; 3 Grupo Boticário, Research and Development Department, R. Alfredo Pinto, 198, Afonso Pena, São José Dos Pinhais, PR Brazil

**Keywords:** Biochemistry, Biotechnology

## Abstract

The continuous search for natural products that attenuate age-related losses has increasingly gained notice; among them, those applicable for skin care have drawn significant attention. The bioester generated from the *Chenopodium quinoa’s* oil is a natural-origin ingredient described to produce replenishing skin effects. With this as motivation, we used shotgun proteomics to study the effects of quinoa bioester on human reconstructed epidermis tridimensional cell cultures after 0, 3, 6, 12, 24, and 48 h of exposure. Our experimental setup employed reversed-phase nano-chromatography coupled online with an Orbitrap-XL and PatternLab for proteomics as the data analysis tool. Extracted ion chromatograms were obtained as surrogates for relative peptide quantitation. Our findings spotlight proteins with increased abundance, as compared to the untreated cell culture counterparts at the same timepoints, that were related to preventing premature aging, homeostasis, tissue regeneration, protection against ultraviolet radiation and oxidative damage.

## Introduction

Skin is the largest human organ and is our first line of defense from the outside world, shielding against ultraviolet (UV), pollution, bacteria, and even viruses. Another of its critical functions is in controlling moisture loss that is ultimately linked to hydration balance. Aside from that, a healthy skin provides a jovial appearance that, in the end, nurtures positivity and overall well-being. In this regard, the World Health Organization (WHO) emphasizes that proper skincare profoundly impacts healthy aging^1^.

Alterations such as reduced levels of lipids, natural moisturizing factors and water have been associated with accelerating skin deterioration and health^2^. Exposure to the harsh external environment on a daily basis has also been linked to skin suffering, especially in its outermost layer, the epidermis. Keratinocytes are the most abundant cell type in the epidermis; they are generated in the basal layer and undergo a process of differentiation, maturation, and then migrate to the surface forming the layers of stratum spinosum, stratum granulosum, and stratum corneum; these layers serve as a permeability barrier and are responsible for aforementioned skin benefits^3^. Dermis containing fibroblasts, its main cell type, represents the inner skin layer and supports epidermal maintenance and differentiation^4^.

Several ingredients of natural origin have been reported to produce nourishing effects on keratinocytes^5^. Some examples are: hyaluronic acid, karite butter, and natural oils that increase the water content and adhesion of the corneocytes in the stratum corneum, keeping it flexible and hydrated^2^. Occlusion in the stratum corneum using oils and oil-based moisturizers has also proven beneficial as it improves homeostasis and maintains the adhesion indices of the corneocytes^6^.

Among the natural ingredients, those from oil origin have gained increasing attention ^7^ In particular, oil from the *Chenopodium quinoa* seeds, when submitted to transesterification, produces a bioester with moisturizing and antioxidant effects^8^. Quinoa contains polyphenols, essential fatty acids, and proteins^9–11^ that exert important bioactivities for the skin^12^. The quinoa has a large amount of 20-hydroxyphysone^13^ that demonstrated to improve dermal thickness^14^, promote wound healing in vivo^15^, increase the differentiation of keratinocytes^16^, and inhibit collagenase activity in vitro^17^. In addition, polyphenols, which make up quinoa-like glycosides, quercetin and kaempferol (flavonoids)^18^, are known to absorb ultraviolet radiation (UV)^19^ and serves as an anti-oxidant^20^. Essential fatty acids have been shown to regulate several cell signaling pathways involved in skin inflammation, dehydration, and tissue degradation^21^. To date, the effects of quinoa derivatives have been investigated only through DNA and mRNA sequencing^12,22^.

Proteomics has served at the forefront as a tool for the development and evaluation of innovative cosmetic products^23,24^. Here, we investigated the proteomic alterations in human epidermis tridimensional (3D) cell culture (RHE), co-cultivated with dermal fibroblasts, after 0 h, 3 h, 6 h, 12 h, 24 h, and 48 h of exposure to quinoa bioester (QB); comparisons were performed with unexposed counterparts at the same time points. The results allowed us to draw important conclusions about how this potent ingredient provides beneficial effects, at the protein level, and pinpointed key proteins related to homeostasis and other key beneficial epithelial tissue functions.

## Results and discussion

Wrinkles, sagging, spots, and tissue dehydration are associated with skin aging and are aggravated without proper care habits such as frequent use of cosmetics and a healthy diet. Skin health impacts the aesthetic appearance and quality of life; unhealthy skin may lead to the development of dermatoses, itching, depigmentation, fungal or bacterial infections^25^. Natural ingredients serve as a treasure trove to form the basis of new cosmetics^24^. The *Chenopodium Quinoa* oil, evaluated in this study, is rich in essential fatty acids, minerals, and amino acids; these are known to be highly emollient and replenishing to the skin. In addition, quinoa oil is a source of antioxidant tocopherols and a potent anti-inflammatory complex that helps replenish the barrier function of the epidermis and prematurely combat tissue damage^26^. With this as motivation, we performed a time-course experiment to verify the effects QB on the epidermis in vitro^27^. We opted for the Reconstructed Human Epidermis (RHE) cell culture as this system mimics the in vivo 3D structure of epidermal tissue as well as the conditions and processes that occur in exposure to exogenous factors^23^.

### Microscopy assessment of RHE differentiation

**Figure 1:**
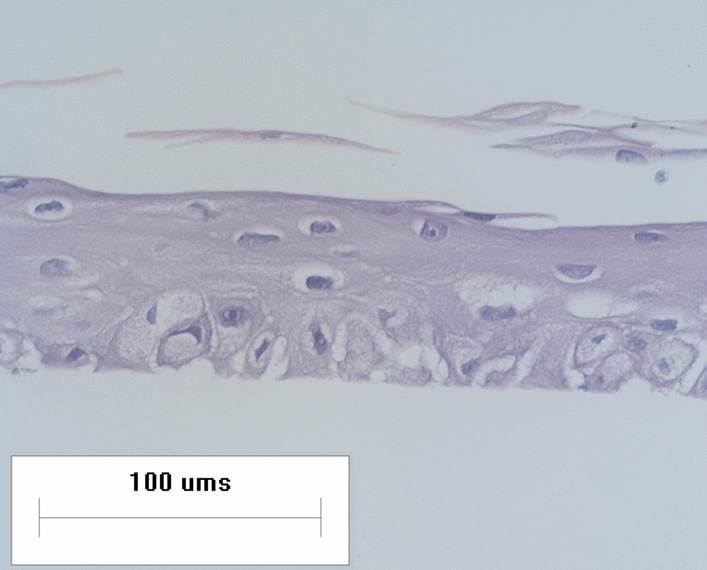
Reconstructed human epidermal (RHE) histology image. Hematoxylin and eosin stain. Objective lens amplification of 20 × were used.

Keratinocyte migration time from basal layer to stratum corneum was evaluated through phase-contrast microscopy. In accordance with previous results, our results show that it is possible to observe the complete differentiation of epidermal layers after 15 days of in vitro culture (Fig. 1)^28,29^.

### Proteomic identifications

Our proteomic analysis identified up to 3981 proteins with 32,897 peptides. The complete list of identified proteins is found in Supplementary Table S1. The list of statistically differentially proteins for 3 h QB-treated vs 3 h QB-free, 6 h QB-treated versus 6 h QB free, 12 h QB-treated versus 12 h QB-free, 24 h QB-treated versus 24 h QB free, and 48 h QB-treated versus 48hQB free are listed in Supplementary Tables S2–S6, respectively.

### Usage of T0 as experimental quality control for our bioinformatics pipeline

**Figure 2:**
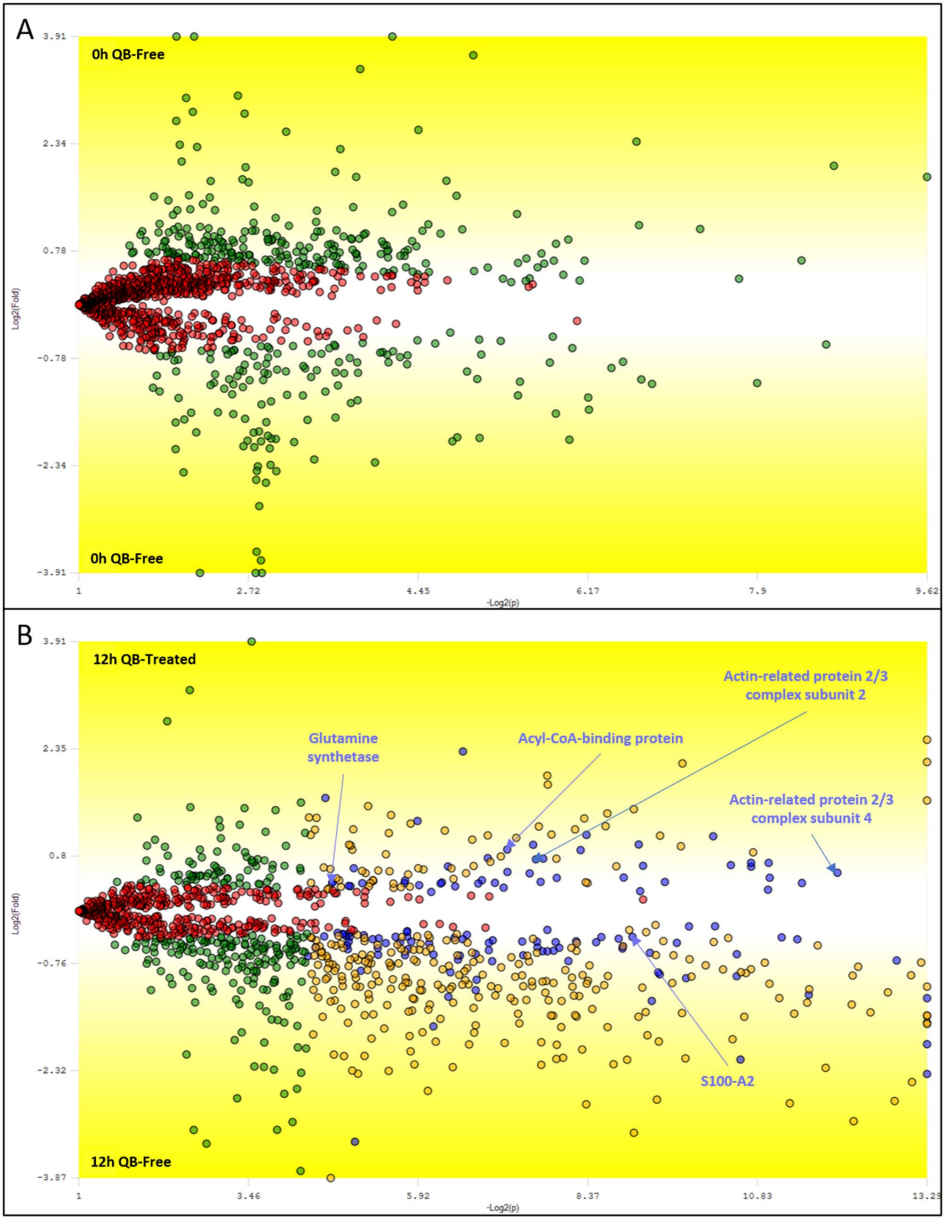
Volcano plot generated with PatternLab’s TFold module comparing 3D cell cultures of keratinocytes. The software parameters were Benjamin Hochberg q-value (FDR) of 0.05, F-Stringency 0.10, and L-Stringency 0.60. Each dot represents a protein that is mapped according to its − log2(P-value) (x-axis) and log_2_(fold change) (y-axis). Red dots are proteins that do not satisfy the fold change cutoff and the q-value cutoff. Green dots are proteins that satisfy the fold change but not the q-value cutoff. Orange dots are proteins that satisfy both the fold change cutoff and q-value cutoff but received very low quantitative values and therefore were disregarded from the analysis. Finally, the blue dots are proteins that satisfy all statistical filters and the ones we consider as statistically differentially abundant. (**A**) Comparison of groups of 3D cell cultures not exposed to QB. Red dots count: 1002. Green dots count: 381. Orange dots count: 0. Blue dots count: 0. (**B**) Comparison of groups of 3D cell cultures exposed versus not exposed to QB after 12 h. Red dots count: 469. Green dots count: 256. Orange dot count: 321. Blue dot count: 127.

The 0 h time-point (T0) of our cell culture was used to verify the effectiveness of our experimental and computational pipeline as more biological replicates were acquired. The assessment for chromatographic reproducibility of technical replicates was done using RawVegetable (Supplementary Fig. S1)^30^. It is expected that no (or almost no) statistically differentially abundant proteins should be identified when comparing groups of biological replicates from the same biological condition. As expected, no differential proteins were shortlisted in PatternLab’s T-Fold analysis (Fig. 2A). In contrast, all other timepoints listed differential proteins when comparing the QB-treated cell culture with its QB-free counterpart for the same timepoint. Figure 2B demonstrates a TFold volcanoes plot comparing QB treated versus the QB-free cells after 12 h; a comparison for all time points is available as Supplementary Figs. S2–S7.

### QB induces Acyl-Coa that is linked with homeostasis

Acyl-Coa is part of the Fatty acid metabolism pathway. The stratum corneum (SC) serves as a barrier between the deeper layers of the skin and the external environment and controls homeostasis^31,32^; improper hydration impairs certain enzymatic functions resulting in the adhesion and accumulation of corneocytes^33^. The SC is commonly described as the skin’s brick-and-mortar, where the anucleate corneocytes, mainly composed of keratin, are within a matrix rich in lipids containing cholesterol, ceramides, esters, and fatty acids^34^. The Acyl-CoA protein (ACBP) binds to esters with high specificity and affinity and acts as an intracellular carrier in various enzymatic systems. ACBP is abundant in the epidermis, the suprabasal layers, which are highly active in lipid synthesis. According to Bloksgaard et al*.*, the silencing of the ACBP gene in mice caused oiliness, development of alopecia, and skin scaling. Moreover, it compromised the function of the epidermal barrier, causing an increase in the loss of transepidermal water, indicating this consequence of reduced levels of non-esterified fatty acids in the stratum corneum^29^. Our results demonstrate QB stimulates Acyl-Coa after 3 h (59%), 6 h (62%), 12 h (85%), and 24 h (99%) (p < 0,01), according to the TFold analyses.

### QB stimulates glutamine synthetase that has been linked with skin regeneration

Glutamine synthetase is part of the metabolism pathway and is the only enzyme able to catalyze the synthesis of Glutamine (Gln) from ammonia and glutamate; this amino acid is essential for various tissue functions. GS plays an essential role in the acid–base balance and is used as an energy source. In cellular division, it acts as a precursor for the synthesizing of several biologically active compounds, such as purines and pyrimidines^35^. A deficiency of glutamine leads to maleficent responses such as the appearance of erythema and the formation of blisters on the tegument^36,37^. The importance of glutamine in the role of recovery from burn injuries has also been described^38^. GS plays key roles throughout the various layers of the epidermis: in the basal layer, for housekeeping the keratinocyte accumulation cells and in the stratum corneum as a physical and chemical barrier against UV and pollution^31^. GS also controls the homeostasis in the epidermis^39^, provides tissue resistance, and reduces the paracellular permeability^40^. Our results showed that QB favors an increase in the abundance of GS in our 3D cell culture after 3 h (26%), 6 h (38%), and 12 h (34%) (p < 0.01), according to TFold analyses for all time points.

### QB stimulates Actin-related protein 2/3 subunits 2 and 4 that are linked with epidermal morphogenesis and homeostasis

The Actin-related protein 2/3 (Arp 2/3) is part of the EPH-Ephrin signaling pathway. Skin barrier alterations, hyperproliferation, and epithelial hypertrophy are characteristic of epidermal homeostasis changes, leading to different diseases, such as psoriasis^41,42^. The actin-related protein (Arp2/3) complex consists of two actin-related proteins and five additional actin-associated protein complex subunits (Arpc1-5); Arpc2 and Arpc4 as a core subunit. The Arp2/3 complex regulates actin-associated processes, such as endocytosis, cell migration, vesicle trafficking, organelle remodeling, and cell–matrix and cell–cell adhesion^43^. Studies have shown that the downregulation of the Arp2/3 complex in mouse epidermis causes interference in morphogenesis and homeostasis^44^. Our data analysis showed that QB increased the abundance Arpc4 after 3 h (24%), 12 h (47%), and Arpc2 in 12 h (68%) and 24 (63%) with *p* < 0.01, thus suggesting that it could be beneficial for those influences epidermal morphogenesis and homeostasis, according to TFold analyses for all time points.

### Cellular retinoic acid-binding protein-II (CRABP-II) is more abundant in QB-exposed cells and has been linked to preventing premature aging

The CRABP-II is part of the retinoic acid signaling pathway. Skin aging is classified into extrinsic aging, by environmental exposure, such as UV radiation and intrinsic determined by genetic factors^45^. CRABP-II is expressed by suprabasal fibroblasts and keratinocytes and defines a family of proteins that bind to all-trans-retinoic acid (atRA)^46^. atRA’s have a profound effect on the growth and differentiation of human epidermal cells in vivo and in vitro^47^ playing a crucial role in skin homeostasis^48^, controlling the epithelial width, thickening the epidermal by boosting the proliferation of keratinocytes and thus serving as UV protection^49^ and ultimately for preventing carcinogenesis^50^.

The biologically active form of retinoic acid is vitamin A, also known as retinol (ROL), a precursor of retinoic acid. Human skin can convert ROL into its biologically active retinoic metabolite. When used topically on human skin, ROL permeates it, becomes converted to retinaldehyde and then to retinoic acid^51,52^. The signaling of retinoic acid (RA) is essential for epidermal differentiation^53^. The regulation of intracellular retinoid bioavailability is made by the presence of specific retinol and retinoic acid-binding proteins, such as CRAPBS^50^. Our results showed that QB increases the levels of the cellular retinoic acid-binding protein-II; effects were especially notable after 48 h presenting an increase in abundancy of ~ 50% (*p* < 0.01), according to the TFold analyses for all time points.

### QB induces downregulation in S100-A2 that is linked with oxidant defense

S100 proteins belong to a family of cytosolic calcium-binding proteins, composed of 25 members^54^ with different intracellular and extracellular functions. The S100A2 protein is located in the basal layer of the human epidermis^55^, having its overexpression in epidermal dysfunctions of morphogenesis and homeostasis^56^. According to Zhang et al*.*, downregulation of S100-A2 is associated with defense against oxidants in epithelial tissue^57^. Our results demonstrate the S100-A2 downregulation at 3 h (15%) and 12 h (29%) (*p* < 0.01), according to the TFold analyses.

### Time-course analysis suggests that QB favors cornification

**Figure 3:**
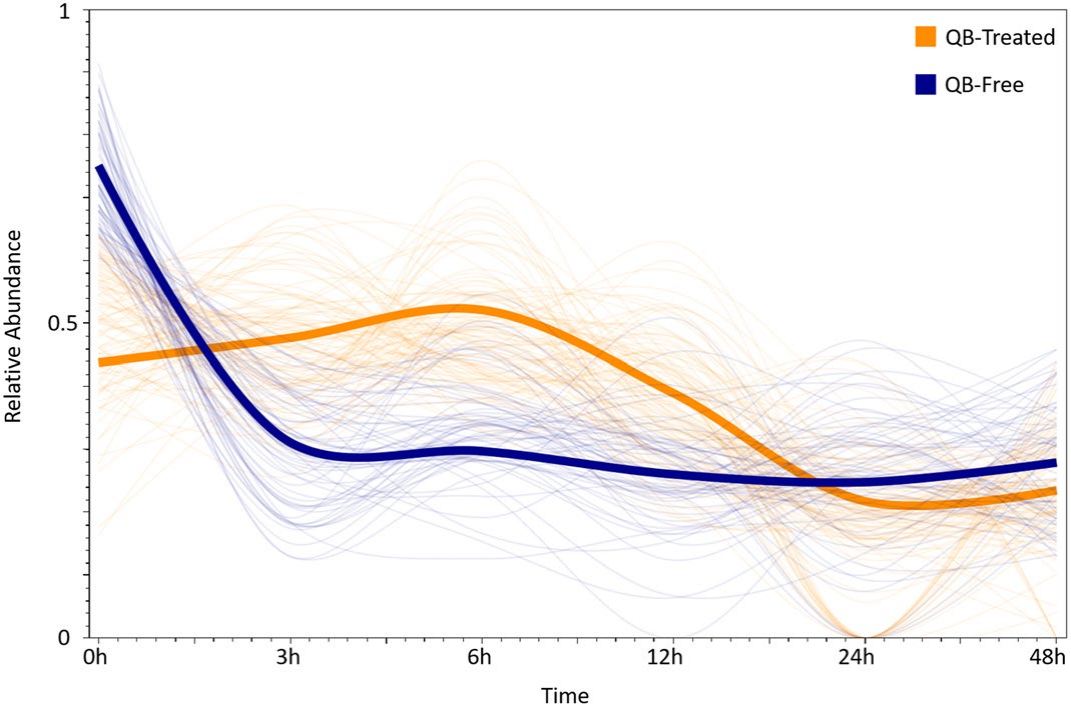
TrendQuest analysis. Proteins with similar abundance profiles were grouped. The orange and the blue thick lines represent the normalized average protein profile for the QB-treated and QB-free clusters for the “Translation” and the “Formation of the cornified envelope” pathways, respectively. The thin lines derive from individual proteins from the corresponding clusters.

**Figure 4:**
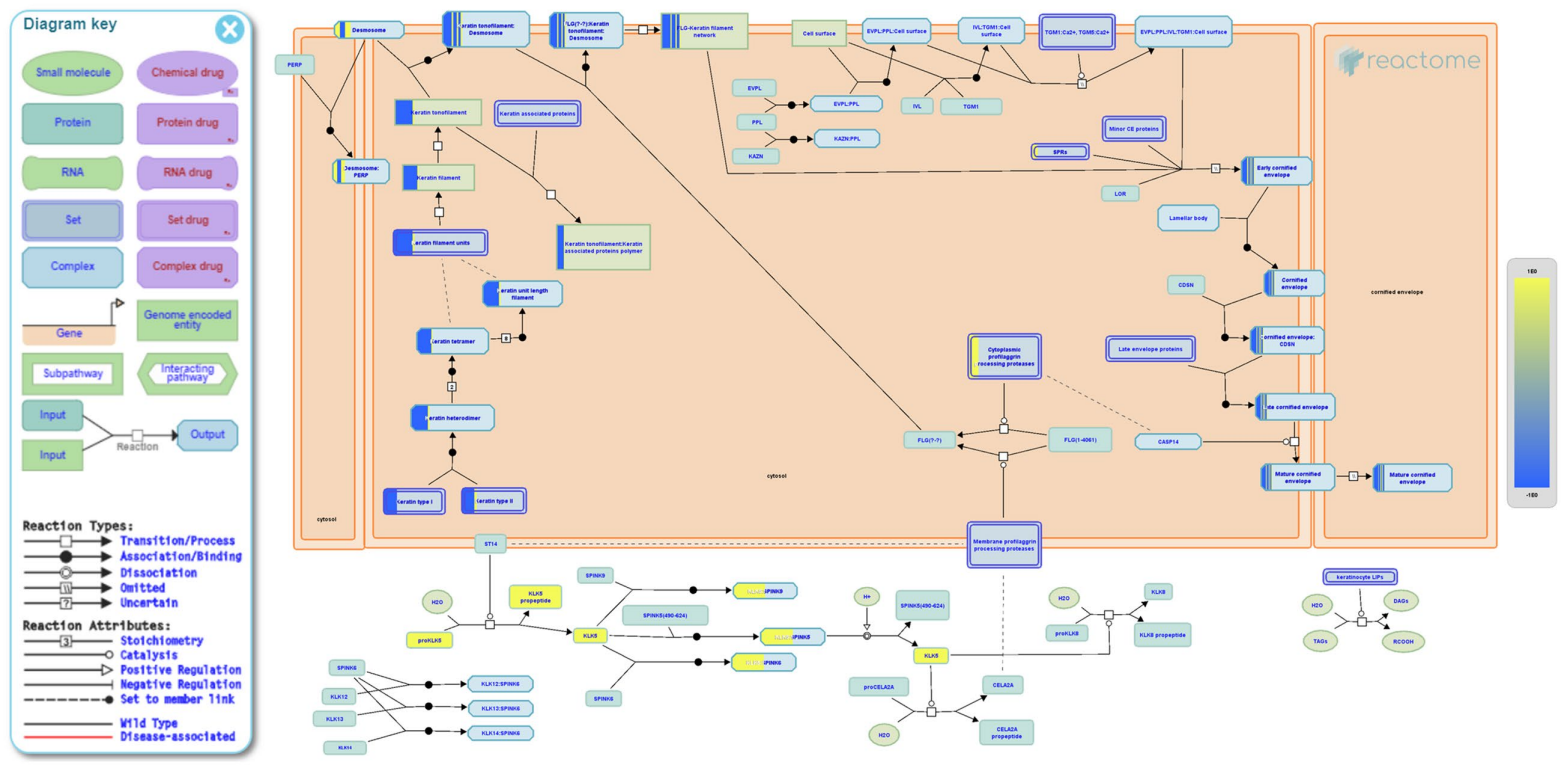
Formation of the cornified envelope pathway Reactome analysis. The left panel presents the Reactome’s legend; Pathway Diagram shows compartments: the big orange box representing the cytosol, bounded by a double-line representing the plasma membrane, and the white background outside the box represents the extracellular spaces. The intracellular diagram represents the enriched pathways. Yellow proteins and blue proteins originate from the QB-treated and the QB-free cell culture trends, respectively.

PatternLab’s TrendQuest module was applied to group proteins that shared a similar abundancy profile over our time-course experiment. The software converged to five clusters for QB-Free and another five for QB-Treated cells. Only proteins found in three or more time points were considered. Table 1 provides a bird’s-eye view for all clusters; each one is presented side-by-side with its most enriched pathway. Supplementary Table S7 includes detailed information and plots for all clusters. In general, the enriched pathways suggest that QB favors cornification. Cornification refers to the formation of a dead cell (corneocyte) layer that serves as a protective physical barrier for the skin^58^. Several pathways are activated during homeostatic keratinocyte differentiation to control the keratinocytes from premature apoptosis and necrosis to enable the keratinization process^59,60^. Among the enriched pathways displayed in Table 1, we highlight the “The citric acid (TCA) cycle and respiratory electron transport” (Table 1—Q4) and the “Cholesterol biosynthesis” (Table 1—Q5) obtained from the QB treated cells. The former (Q4) is intimately related to the stratification process and the later (Q5) with the permeability barrier formation. Stratification begins with the downregulation of adhesion molecules, subsequent detachment of the basal cells of the basement membrane, and migration of the part of the innermost layer (basal) to the outermost layers (suprabasal), developing the component layers of the tissue^61^. Biosynthesis of cholesterol and other lipids in the skin is responsible for the epidermal permeability barrier and is another essential and desired quality for promoting homeostasis. Our results demonstrate that both pathways were triggered after 12 h, suggesting that QB favors the renewal of essential elements to enable homeostasis and the integrity of the skin barrier. In contrast, these pathways were far from topping the list in the QB-Free cell line. In fact, in QB-free cells, the “Formation of the cornified envelope” pathway (Table 1—F4) decreases over time as the “Methylation” (Table 1—F3) pathway increased. Such an inverse correlation is well described in the literature; increased methylation suppresses the differentiation and maintains cell proliferation at baseline levels^62^. Figure 3 contrasts profiles from clusters Q3 and F4; a joint Reactome analysis (Fig. 4) shows that both are related to the cornification process and yet, in our results, their general abundancy trend is indirectly correlated. Finally, we note a common enriched pathway to both QB-free and QB cell-lines: “Formation of the ternary complex, and subsequently, the 43S complex” (Table 1, Q1 and F1). The aforementioned pathway is found in clusters that share a similar protein profile distribution for both the QB and QB-free cells; it is related to essential tasks and thus remains with its relative quantitation mostly unaltered throughout.

**Table 1:** Top enriched pathways per cluster.

Node	Pathway identifier	Pathway name	#Entities found	#Entities total	Consensus
Q1	R-HSA-72695	Formation of the ternary complex, and subsequently, the 43S complex	44	52	
Q2	R-HSA-72731	Recycling of eIF2:GDP	4	8	
Q3	R-HSA-72766	Translation	17	294	
Q4	R-HSA-1428517	The citric acid (TCA) cycle and respiratory electron transport	12	176	
Q5	R-HSA-191273	Cholesterol biosynthesis	2	26	
F1	R-HSA-72695	Formation of the ternary complex, and subsequently, the 43S complex	40	52	
F2	R-HSA-72766	Translation	32	294	
F3	R-HSA-156581	Methylation	3	14	
F4	R-HSA-6809371	Formation of the cornified envelope	13	129	
F5	R-HSA-163200	Respiratory electron transport, ATP synthesis by chemiosmotic coupling, and heat production by uncoupling proteins	9	125	

The “Node” column presents Q1–Q5 and F1–F5 for clusters of protein profiles obtained from the quinoa bioester treated and from the Quinoa Free cell lines, respectively. The “Pathway Identifier” and “Pathway Name” columns refer to the corresponding entries per Reactome. The “Entities Found and “Entities Total” is the number of proteins identified in this study belonging to the respective pathway and the total number of proteins cataloged for that pathway, as per Reactome, respectively. Finally, the “Consensus” column represents the consensus protein profile of all identified proteins belonging to the respective cluster; the *y*-axis representing relative abundancy and the *x*-axis related to the different time points.

## Material and methods

### Material

Human Epidermal Keratinocytes neonatal (HEKn) (cat. no. nh-skp-KT0069) were obtained from *Banco de Células do Rio de Janeiro* (BCRJ). Human Dermal Fibroblasts neonatal (HDFn) (cat. no. C0045C) and Penicillin–Streptomycin (P/S) (cat. no. 15070063) were acquired from Thermo Fischer. Phosphate Buffered Saline (PBS) pH 7.4 (cat. no. 10010031), Dulbecco’s Modified Eagle’s Medium (DMEM) and Fetal Bovine Serum (FBS) (cat. no. 12800017) were purchased from Gibco. KBM Gold Keratinocyte Growth Basal Medium (cat. no. 00192151) was purchased from Lonza. Twelve Well Cell Culture Plate Cellstar (cat. no. 66518001) and ThinCert Cell Culture Insert (cat. no.665641) were acquired from Greiner. Trypsin–EDTA (0.25%) and Albumin Bovine Serum (cat. no. 12657) were purchase from Merck. Qubit Protein Assay Kit (cat. no. Q33212) and RapiGest acid-labile surfactant (cat. no. 186001861) were acquired from Invitrogen and Waters, respectively. Sequence grade modified trypsin (V511A) was purchased from Promega.

### Cell culture

The Reconstructed Human Epidermis 3D cell culture (RHE) was adapted from Brohem and co-authors^63^. Although this work focused on evaluating protein expressions by keratinocytes, fibroblasts were co-cultivated in a different experimental compartment to simulate epidermal and dermal communication. Fibroblasts were cultured in DMEM supplemented with 10% FBS and 1% P/S, while keratinocytes were cultured with KBM Gold medium. For subcultures, the confluent monolayers were gently washed with PBS and after brief 3-min trypsinization, the cells were suspended in the complete culture medium. For the formation of monolayers, fibroblasts were cultivated into three 12-well plates. After 3 days, the medium was exchanged, and ThinCert was placed in each well with keratinocytes added to its superior face. Finally, 3 days after, the entire liquid content of the insert and the wells were removed, and then performed at air–liquid interphase filled with 1 mL *Grupo Boticário’s* propriety differentiation medium into plates. The medium was changed every 3 days, during 15 days for epidermal differentiation.

### Quinoa bioester (QB) treatment

QB was diluted in Vaseline to a final concentration of 0.01%. The QB was exposed on the surface of the differentiated epidermis with fibroblasts at the base of the wells. Reconstructed Human Epidermis 3D cell culture following time points: 0 h, 3 h, 6 h, 12 h, 24 h, and 48 h, and then immediately harvested, washed with PBS 1 ×, and stored at − 80 °C for further proteomics analysis. The same procedure was accomplished for differentiated RHE with no exposure to QB. For each time point (exposure or not), three biological replicates were performed, totaling 36 cell cultures. Cultures obtained for time zero in both conditions were used for quality assessment of the posterior proteomic analysis. After treatment, three 12-well plates containing adherent fibroblasts were discarded and only RHE 3D cell cultures were considered for the next steps; one of 0 h time point control was used for histological evaluation.

### Histological evaluation of RHE

Briefly, RHE were fixed in 4% (v/v) of formaldehyde and PBS 7.4 for 2 h. Subsequently, the samples were dehydrated in increasing concentrations of ethanol, diaphonized in Xylol, and included in paraffin. Three micrometers (3 µm) sections of the samples were deposited on positively charged slides (Imunnoslide, Easypath). Then, cuts were then dewaxed in xylene, rehydrated in decreasing concentrations of ethanol, then stained using the panocytic hematoxylin and eosin technique, according to CITOLAB^64^.

Phase-contrast microscopy in an Eclipse TE300 Inverted Microscope was employed to evaluate RHE differentiation, applying × 20 objective lens magnification.

### Sample preparation

RHE proteins were extracted with RapiGest detergent at a concentration of 0.1% according to the manufacturer’s recommendations. According to the manufacturer’s instructions, protein concentrations were determined using the fluorimetric assay from the Qubit platform (Invitrogen). One hundred micrograms of proteins from each sample were reduced with dithiothreitol (DTT) (final concentration of 10 mM) for 30 min, at 60 °C. After being cooled to room temperature, the samples were alkylated with iodoacetamide (final concentration of 30 mM) for 25 min at room temperature, in the dark, and finally digested with sequence grade modified trypsin in the proportion of 1/50 (E/S) for 20 h, at 37 °C.

### Desalting and sample quantification

In due course, the enzymatic reaction was stopped by adding trifluoroacetic (0.4% v/v final) and the peptides were incubated for additional 40 min to degraded the RapiGest. Afterward, the samples were centrifuged at 18.000×*g* for 10 min to remove any insoluble materials. Subsequently, the peptides were quantified using the fluorometric assay—Qubit 2.0 (Invitrogen) according to the manufacturer's recommendations. Each sample was desalted and concentrated using Stage-Tips (STop and Go-Extraction TIPs) according to Rappsillber and collaborator^65^.

### Mass spectrometry analysis

The peptides were subjected to LC–MS/MS analysis with a Thermo Scientific Easy-nLC 1000 ultra-high-performance liquid chromatography (UPLC) system coupled with an LTQ-Orbitrap XL mass spectrometer, as follows. The peptide mixtures were loaded onto a column (75 mm i.d., 30 cm long) packed in house with a 3.2 μm ReproSil-Pur C18-AQ resin (Dr. Maisch) with a flow of 250 nL/min and subsequently eluted with a flow of 250 nL/min from 5 to 40% ACN in 0.1% formic acid and 5% DMSO, in a 180 min gradient^66^. The mass spectrometer was set in data-dependent mode to automatically switch between MS and MS/MS (MS2) acquisition. Survey full-scan MS spectra (from *m/z* 300–2000) were acquired in the Orbitrap analyzer with the resolution R = 60,000 at *m/z* 400 (after accumulation to a target value of 1,000,000 in the linear trap). The ten most intense ions were sequentially isolated and fragmented in the linear ion trap using collisional induced dissociation with normalized energy of 35. Previous target ions selected for MS/MS were dynamically excluded for 90 s. The total cycle time was approximately 3 s. The general mass spectrometric conditions were: spray voltage, 2.4 kV; no sheath and auxiliary gas flow; ion transfer tube temperature 175 °C; collision gas pressure, 1.3mTorr; normalized energy collision energy using wide-band activation mode; 35% for MS2. Ion selection thresholds were: 250 counts for MS2. An activation q = 0.25 and an activation time of 30 ms were applied in MS2 acquisitions. Two technical replicates were acquired for each biological replicate.

### Peptide spectrum matching (PSM)

The data analysis was performed with the PatternLab for proteomics 4 software that is freely available at https://www.patternlabforproteomics.org^67^. *Homo sapiens’* sequences were downloaded on June 6th, 2020 from the Swiss-Prot and then a target-decoy database was generated to include a reversed version of each sequence plus those from 104 common mass spectrometry contaminants. The Comet 2019.01 rev. 5 search engine was used for identifying the mass spectra^68^. The search parameters considered: fully and semi-tryptic peptide candidates with masses between 550 and 5500 Da, up to two missed cleavages, 40 ppm for precursor mass, and bins of 1.0005 m*/z* for MS/MS with an offset of 0.4. The modifications were carbamidomethylation of cysteine and oxidation of methionine as fixed and variable, respectively.

### Validation of PSMs

The validity of the PSMs was assessed using Search Engine Processor (SEPro)^69^. The identifications were grouped by charge state (2 + and ≥ 3 +), and then by tryptic status, resulting in four distinct subgroups. For each group, the XCorr, DeltaCN, DeltaPPM, and Peaks Matches values were used to generate a Bayesian discriminator. The identifications were sorted in nondecreasing order according to the discriminator score. A cutoff score accepted a false-discovery rate (FDR) of 2% at the peptide level based on the number of decoys^70^. This procedure was independently performed on each data subset, resulting in an FDR independent of charge state or tryptic status. Additionally, a minimum sequence length of five amino-acid residues and a protein score greater than 3 were imposed. Finally, identifications deviating by more than 10 ppm from the theoretical mass were discarded. This last filter led to FDRs, now at the protein level, to be lower than 1% for all search results^71^.

### Proteomic data analysis

We quantitated, independently, three biological replicates for each of our six-time points (i.e., T0h, T3h, T6h, T12h, T24h, T48h), with two technical replicates. Quantitation was performed according to PatternLab's Normalized Ion Abundance Factors (NIAF) as a relative quantitation strategy and as described in our bioinformatics protocol^67^. We recall that NIAF is the equivalent to NSAF^72^, but applied to extracted ion chromatogram (XIC). Differentially abundant proteins were listed by using PatternLab’s TFold module to compare time point zero with the other time points^73^. We also performed a TFold analysis comparing the two batches of biological replicates acquired for timepoint 0 h to serve as a quality control step; we expected to find no differentially abundant proteins as all 3D cell cultures originated from the same biological condition. PatternLab’s TrendQuest module was also employed to group proteins that share the same temporal abundancy patterns over the time-course experiment^74^. Finally, we used the Reactome^75^ tools to help interpret the data.

### Conclusion

Here, we pinpointed proteomic alterations that 3D keratinocyte cell cultures undergo when exposed (or not) to QB at several timepoints. Our results shortlisted up-regulated proteins that are known to be beneficial for skin replenishing. We opted for performing our work on 3D cell cultures as they have been described to better mimic in vivo as when compared to 2D cell cultures^76^, thus our results suggest that the application of QB could be beneficial to human skin; nevertheless, in vivo studies should be performed to validate such hypothesis.

## Supplementary information

Supplementary Tables.

Supplementary Figures.

Supplementary Information.

## Data Availability

The mass spectrometry data have been deposited to the ProteomeXchange Consortium via the PRIDE^77^ partner repository with the dataset identifier PXD020893.
