# Supplementary material for: Proteomics reveals that quinoa bioester promotes replenishing effects in epidermal tissue
Source: Sci Rep. 2020 Nov 10;10:19392. doi: 10.1038/s41598-020-76325-6 (PMC7655866; doi:10.1038/s41598-020-76325-6)

**Proteomics reveals that quinoa bioester promotes replenishing effects in epidermal tissue**

Amanda C. Camillo-Andrade^1, a^, Marlon D. M. Santos^2, a^, Juliana S. G. Fischer^2^, Bruna B. Swinka^3^, Bruna Bosquetti^3^, Desirée C. Schuck^3^, Marcia R. Pincerati^1^, Marcio Lorencini^3, *^, Paulo C. Carvalho^2, *^

1 Master Program in Industrial Biotechnology, Positivo University, Curitiba, Paraná, Brazil

2 Laboratory for structural and computational proteomics - Carlos Chagas Institute, Fiocruz Paraná, Brazil

3 Research and Development Department, Grupo Boticário, São José dos Pinhais, Paraná, Brazil

a Equals Contribution

*Corresponding authors

Q1


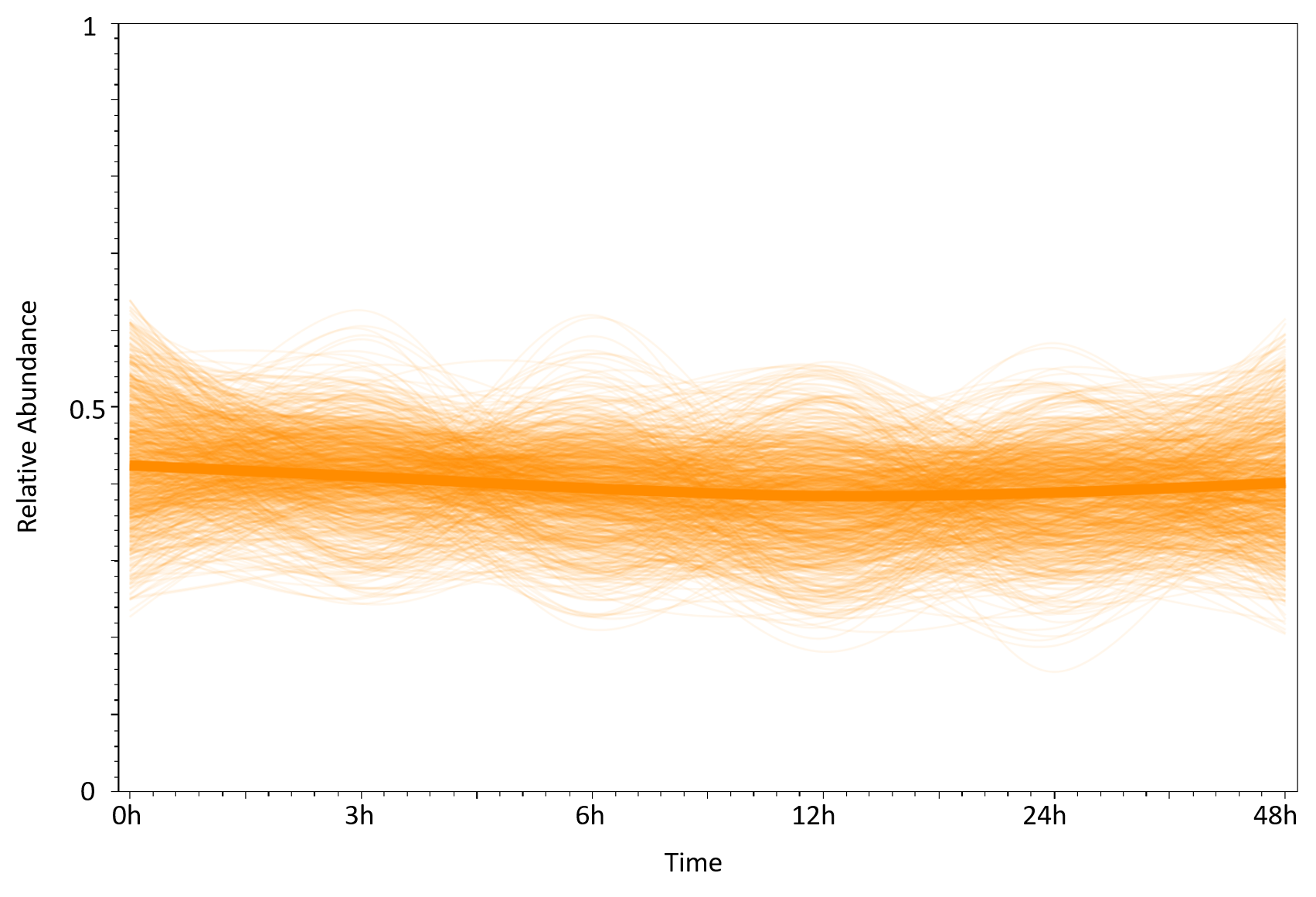


Q2


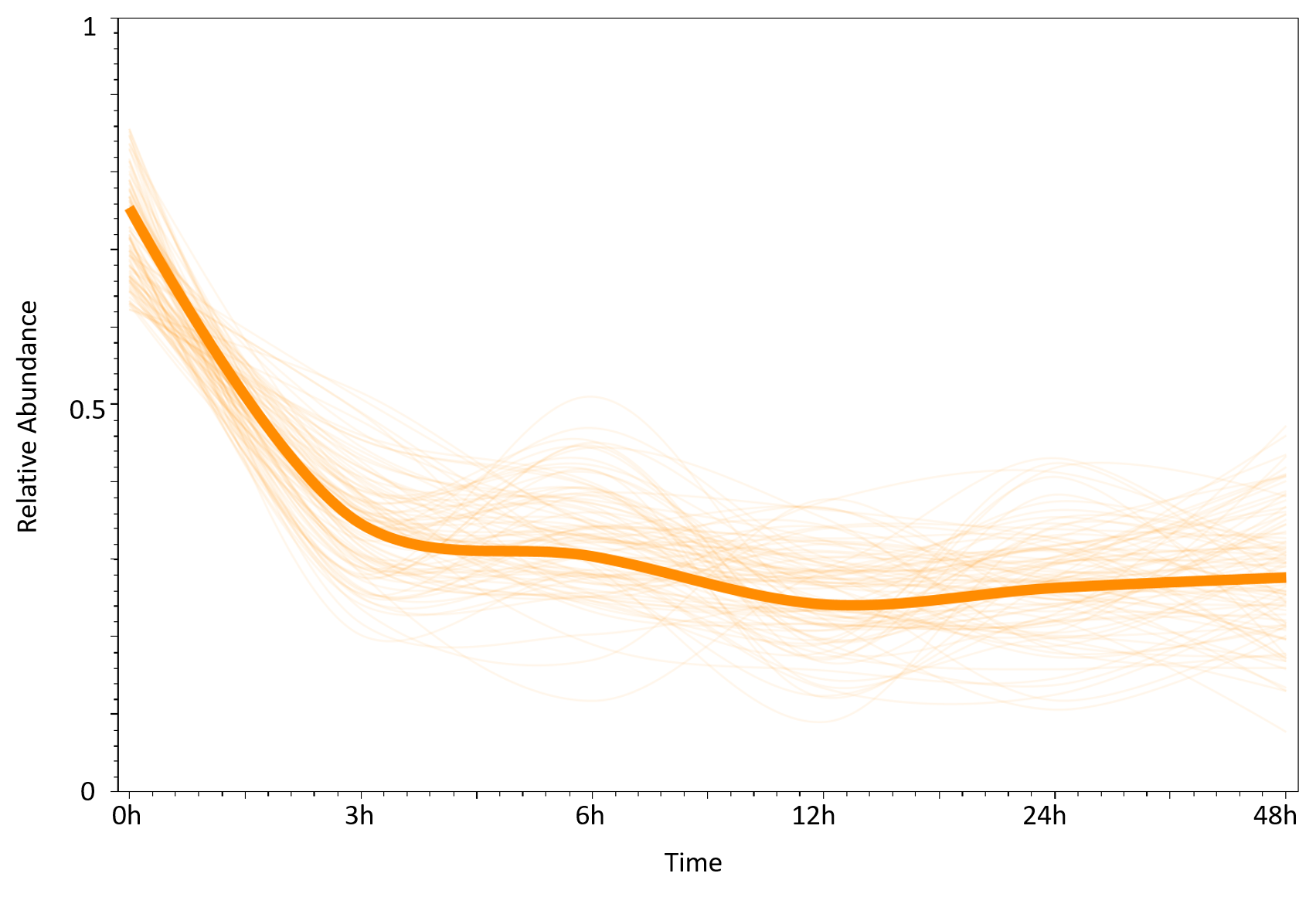


Q3


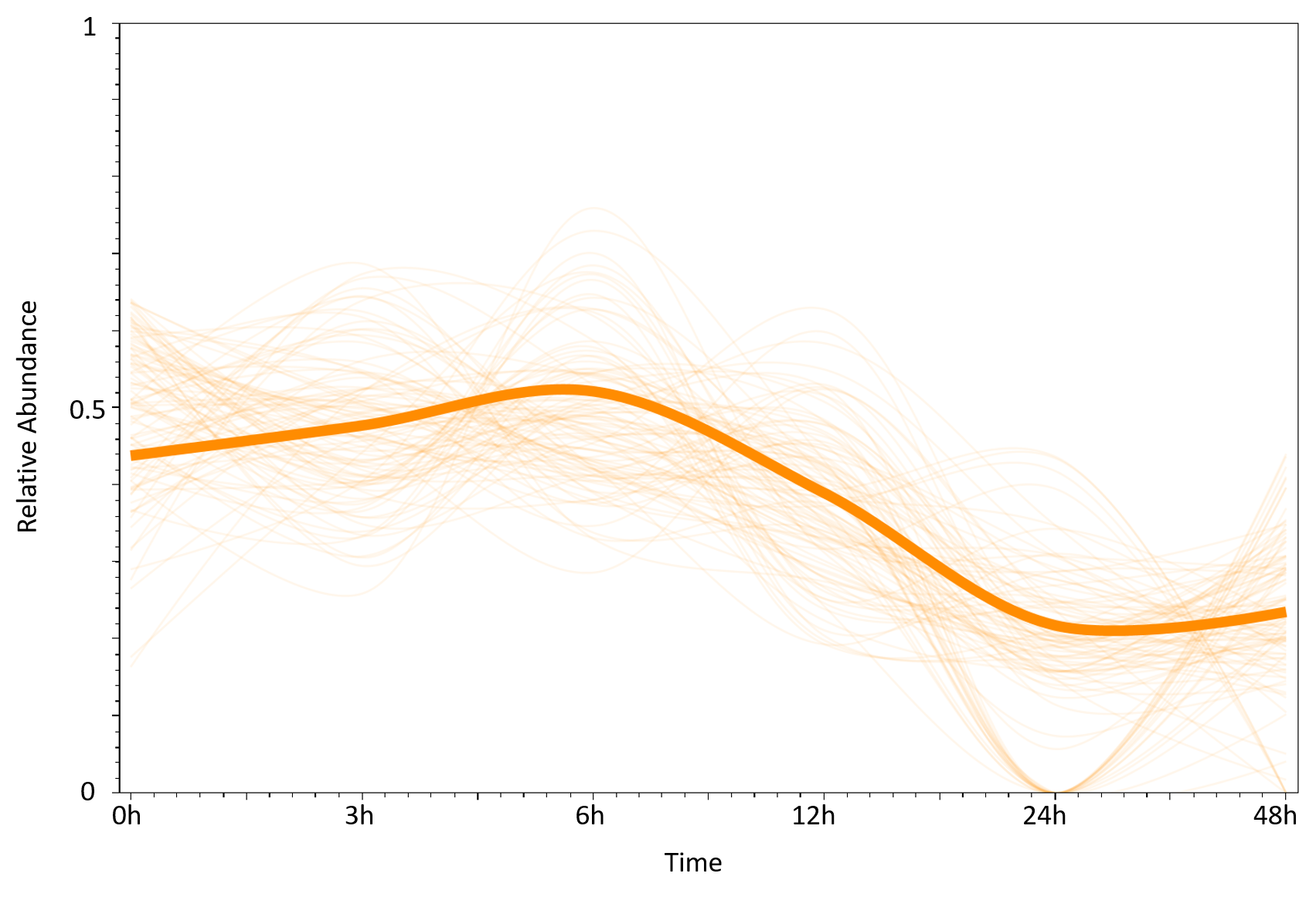


Q4


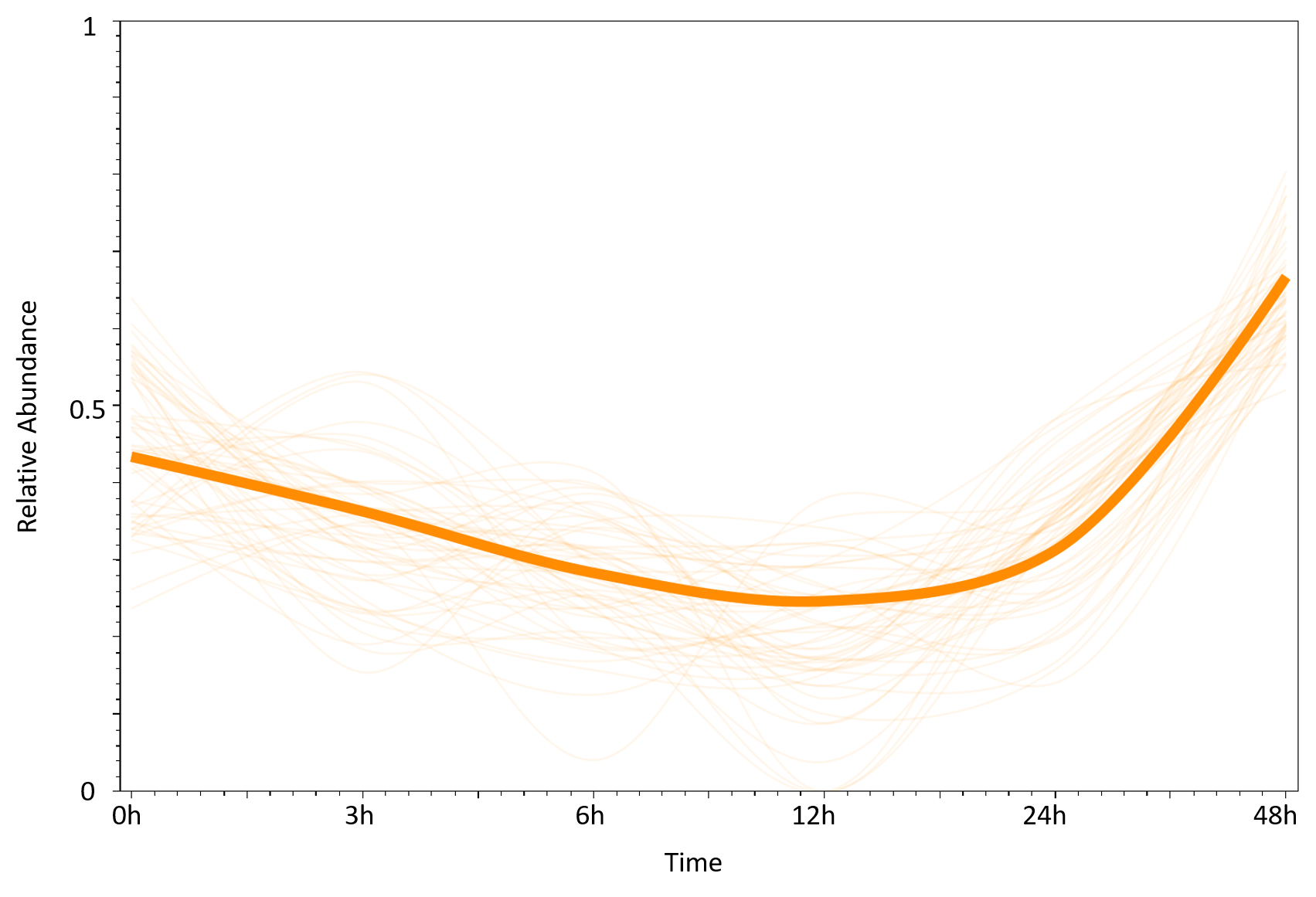


Q5


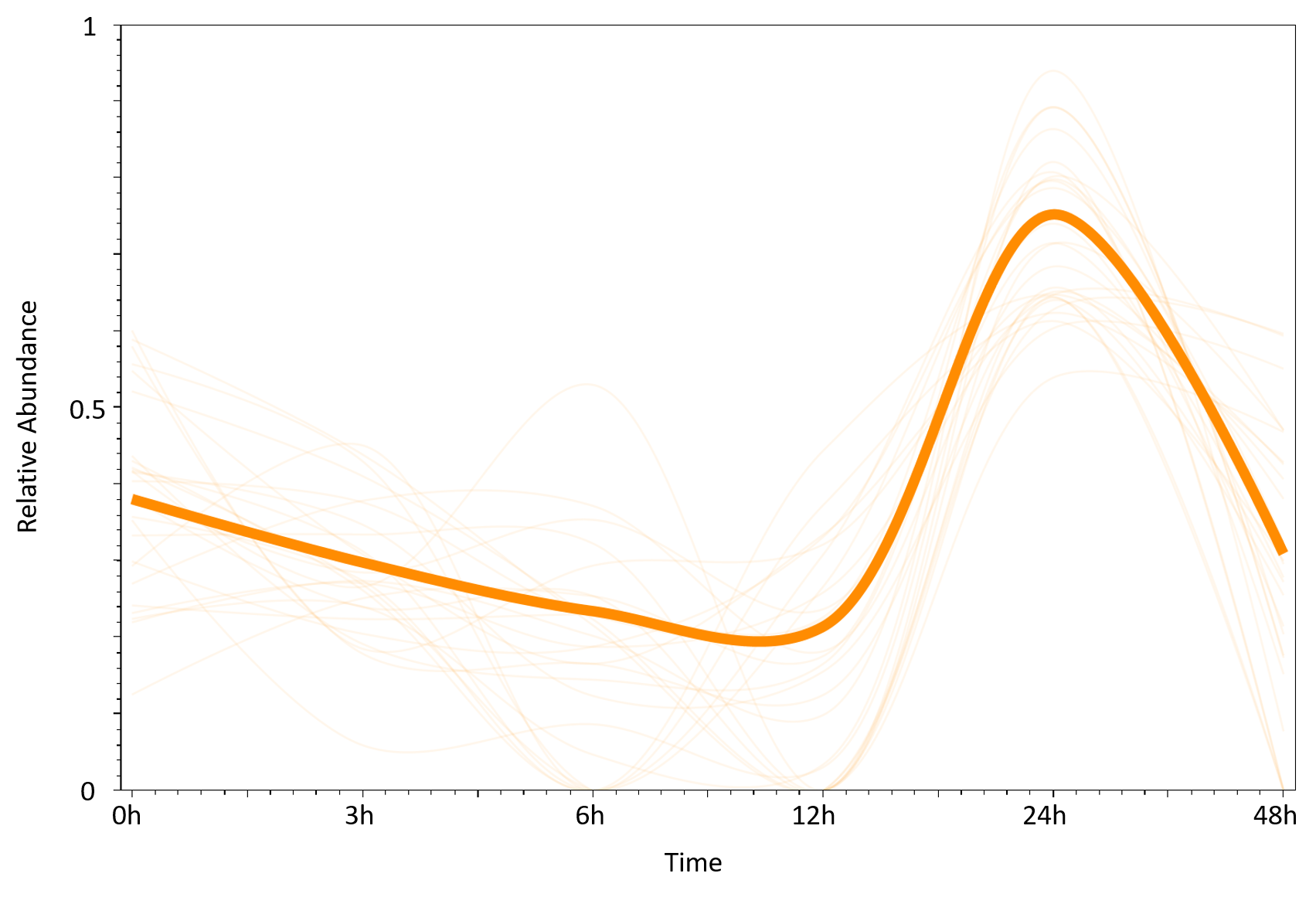


F1


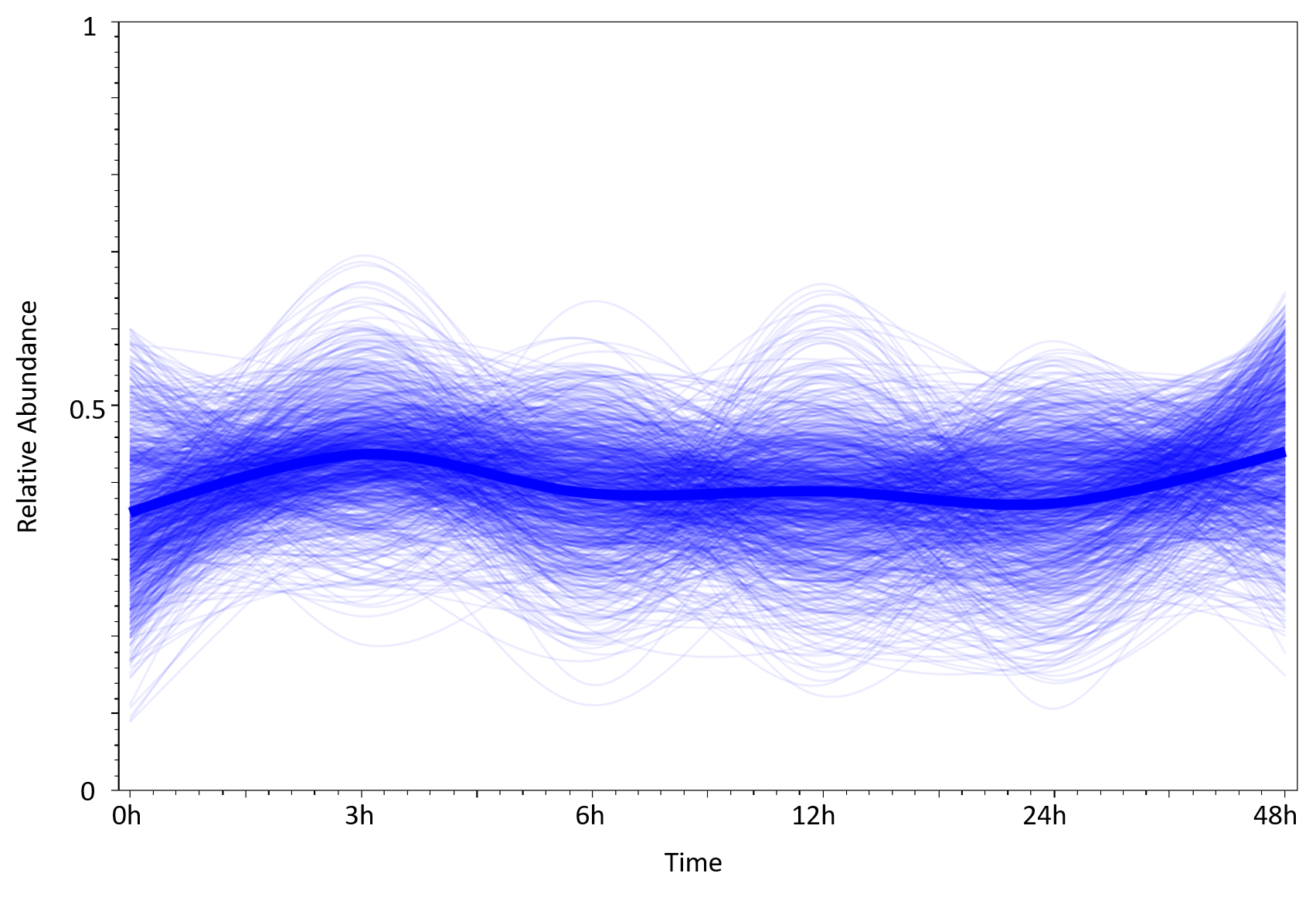


F2


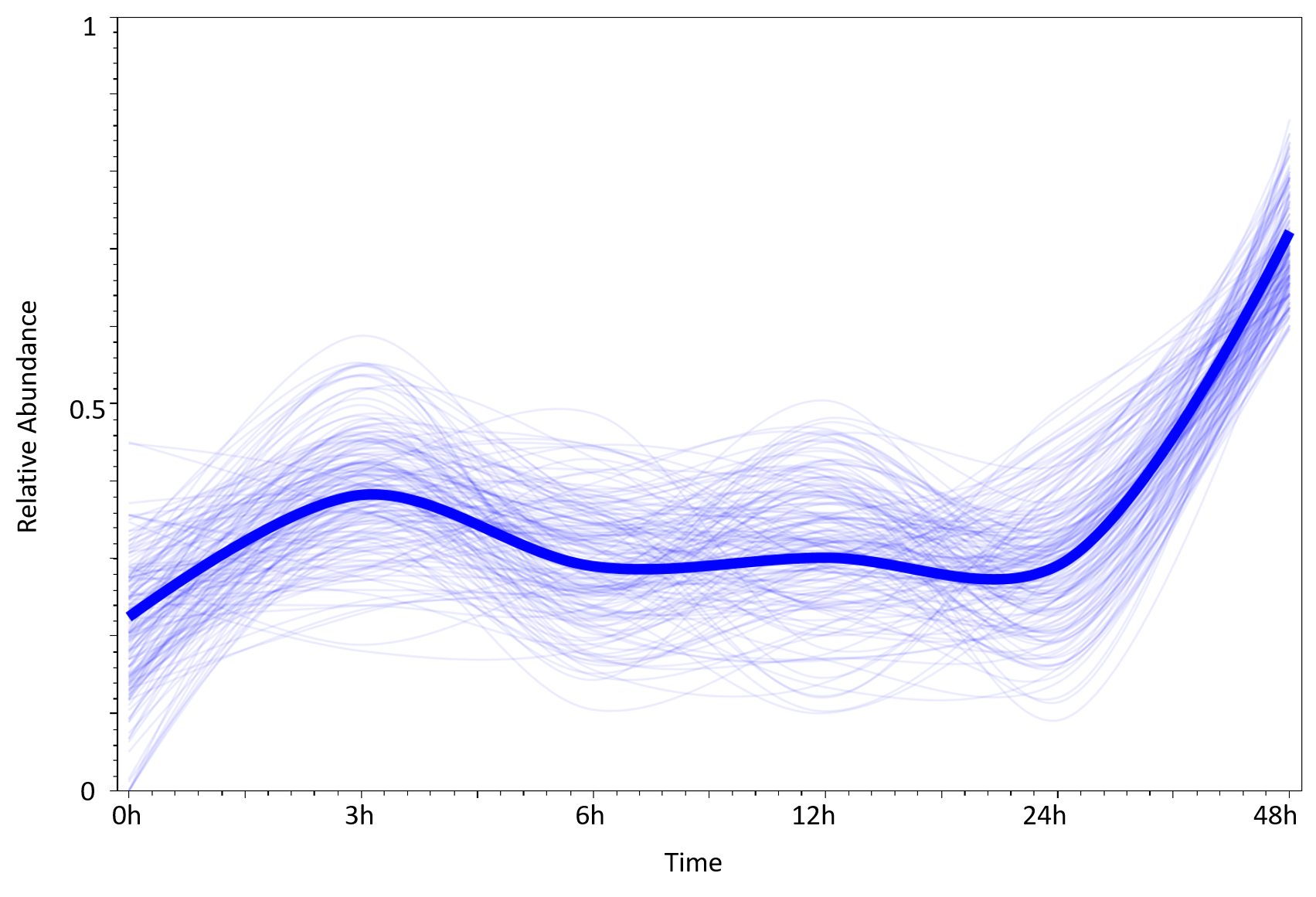


F3


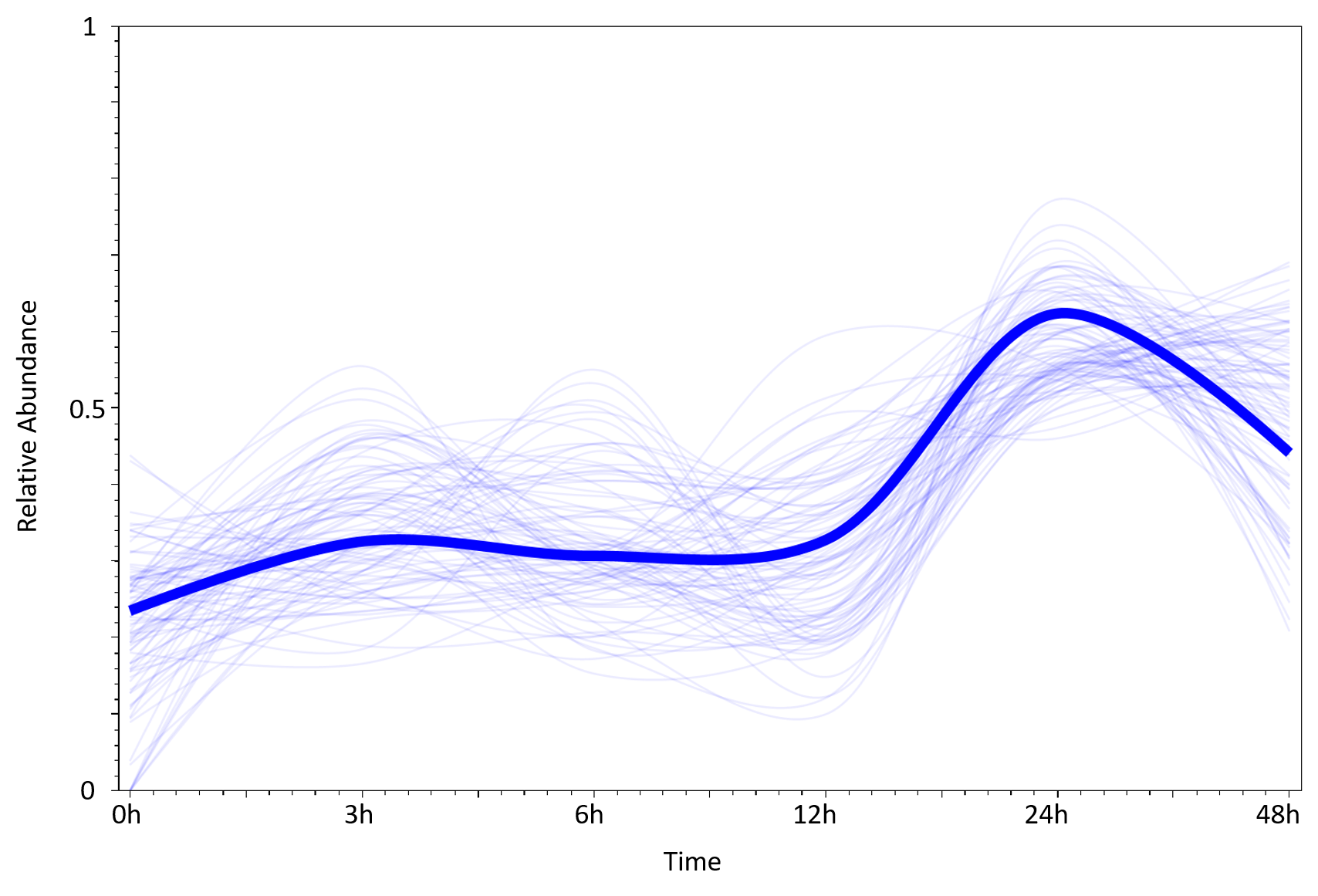


F4


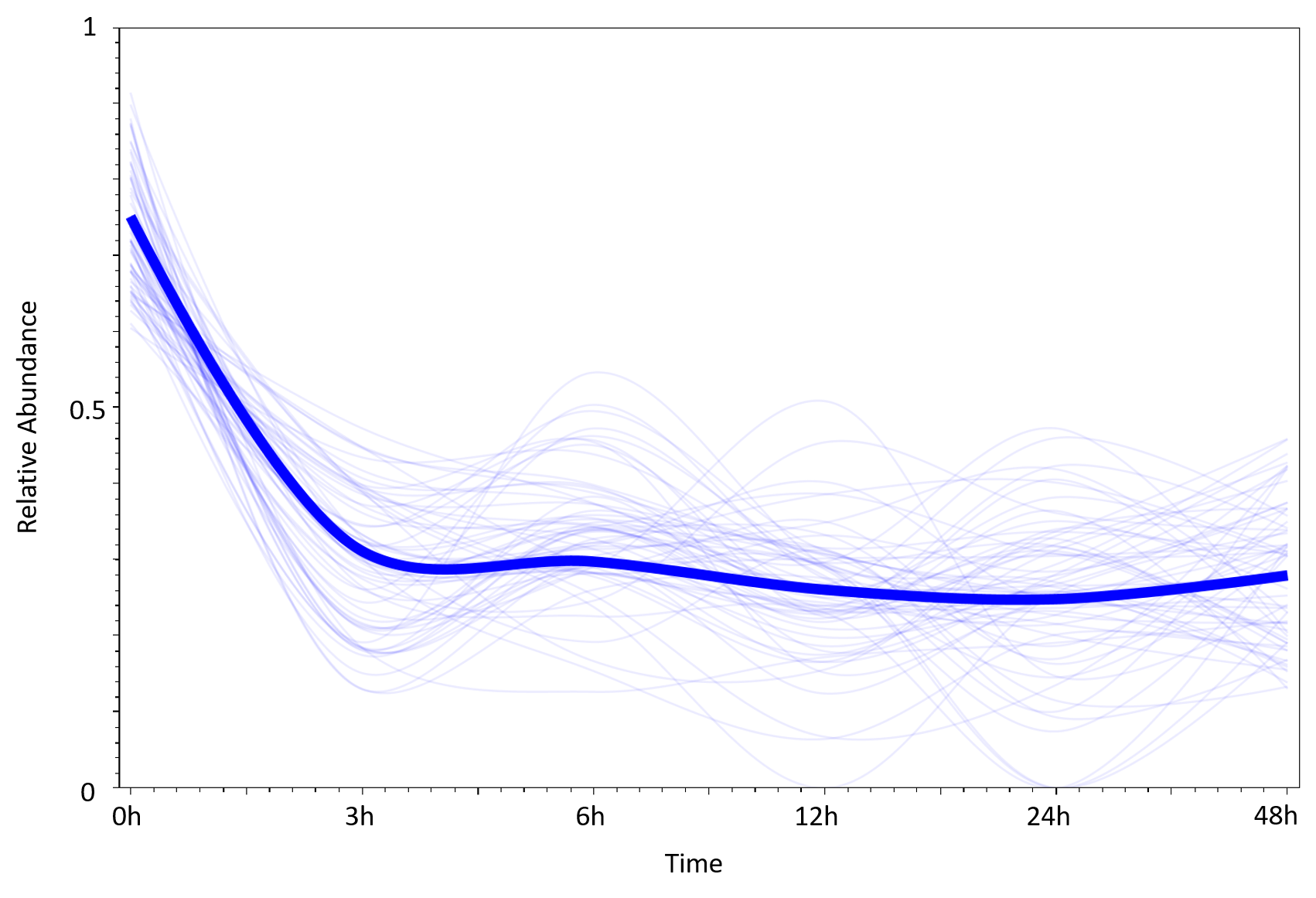


F5


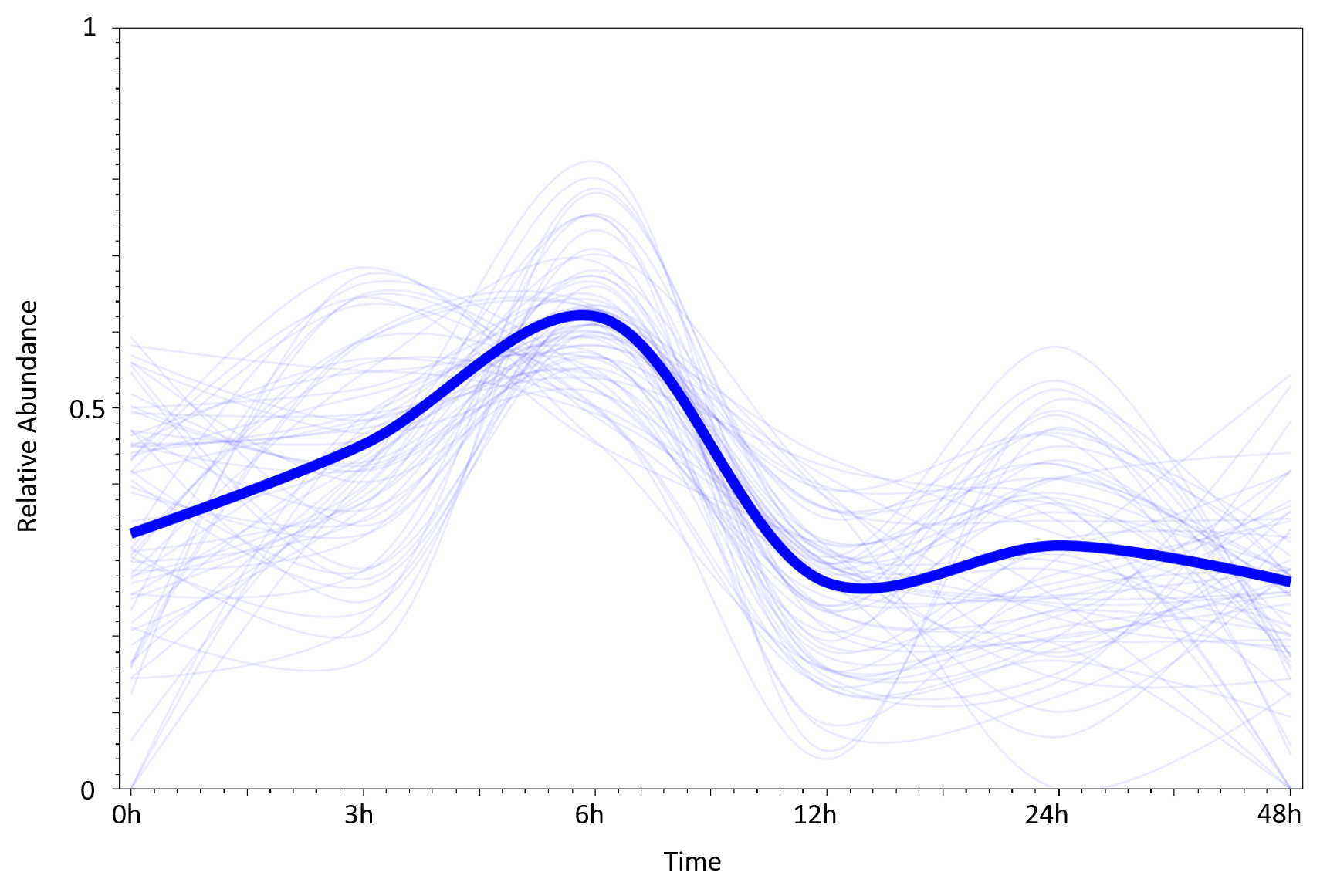

Supplement: Supplementary file 3 — Supplementary Information. [file 41598_2020_76325_MOESM3_ESM.docx]
